# The effect of serum types on Chondrogenic differentiation of adipose-derived stem cells

**DOI:** 10.1186/s40824-018-0116-z

**Published:** 2018-03-09

**Authors:** Hyeran Cho, Aeri Lee, Kyobum Kim

**Affiliations:** Division of Bioengineering, College of Life Sciences and Bioengineering, Incheon National University, 119 Academy-ro, Yeonsu-gu, Incheon, 22012 South Korea

**Keywords:** Fetal bovine serum, Adipose derived stem cell, Chondrogenic differentiation, Serum substitutes

## Abstract

**Background:**

Fetal bovine serum (FBS) is the most essential supplement in culture media for cellular proliferation, metabolism, and differentiation. However, due to a limited supply and subsequently rising prices, a series of studies have investigated a biological feasibility of replaceable serums to substitute FBS. Along with the increasing interests to manufacture stem cell-based cellular products, optimizing the composition of culture media including serums and exogenous growth factors (GFs) is of importance. In this experiment, the effect of bovine serum (BS) and newborn calf serum (NCS) on proliferation and chondrogenic differentiation capacity of human adipose derived stem cells (ADSCs) was evaluated, especially in the chondrogenically supplemented culture condition.

**Methods:**

ADSCs were chondrogenically cultured with FBS, BS, and NCS for 14 days. For the acceleration of in vitro chondrogenesis, exogenous insulin-like growth factor and transforming growth factor-β3 were added. Viability and proliferation of ADSCs were evaluated using Live/Dead fluorescence staining and DNA amount, respectively. To investigate a chondrogenic differentiation, a series of assays were performed including a quantification of glycosaminoglycan deposition, alcian blue staining, and RT-PCR analysis for type II collagen, aggrecan and Sox-9 genes.

**Results:**

The results demonstrated that proliferation of ADSCs was facilitated in FBS condition as compared with other serum types. For chondrogenic marker gene expression, serum substitutes enhanced Sox-9 expression level on day 14. The deposition of glycosaminoglycan was more facilitated in BS condition regardless of additional chondrogenic GFs.

**Conclusion:**

It could be presumably speculated that serum types and exogenous supplements of GFs could also be important parameters to optimize culture media composition, especially in order to maintain the enhanced levels of both proliferation and chondrogenic differentiation of ADSCs during expansion.

## Background

Osteoarthritis (OA) is a common chronic disease among the elderly people or athletes around the world, which could stimulate discomfort and pain due to degradation and inflammation of cartilage tissues [1, 2]. Current OA treatments manage pain, swelling, and joint stiffness and eventually increase joint movement and flexibility [3, 4]. However, these clinical medications have limitations to regenerate damaged cartilage tissues in a persistent manner due to the lack of vasculatures in cartilage. Therefore, the development of a series of cartilage tissue engineering applications has been extensively investigated for sufficient and efficient treatment improving a quality of regenerated cartilage tissues. Since a self-healing procedure of cartilage tissues rarely occurs when damages or defects occur in the joint, alternative treatments using various stem/progenitor cell populations have also been studied [5, 6]. These stem cell approaches target chondrogenic differentiation of delivered or recruited progenitor cells and enhanced deposition of extracellular matrix (ECM) molecules.

Among various progenitor cell populations for engineering cartilage repair, one of the advantages of using adult stem cells is a less invasive isolation procedure from a human donor body without ethical issues, as compared with embryonic stem cells [7]. Adipose derived stem cells (ADSCs) is one of the major adult stem cell sources due to its higher yield and less invasive isolation process, than obtaining mesenchymal stem cells (MSCs) from bone marrows usually in iliac crest. One of the current medical treatments for OA is a transplantation of isolated chondrocytes from the healthy donor site of the patient to the damaged joint site. However, due to the donor site morbidity in a load-bearing joint, this current method is somehow not suitable [8, 9]. One of the current medical treatments for OA is a transplantation of isolated chondrocytes from the healthy donor site of the patient to the damaged joint site. However, due to the donor site morbidity in a load-bearing joint, this current method is somehow not suitable [10, 11] and de novo cartilage formation 6 weeks after implantation in an in vivo sheep model [12]. In addition, recent clinical approaches demonstrated that autologous ADSC could manage OA-related pains [3, 13]. Furthermore, ADSC inhibit progression of OA. However, specific therapeutic agents aiming at fundamental joint repair are still under being investigated [14, 15]. Hence, clinical demands for the process development of ADSC expansion are recently required. Chondrogenesis of transplanted ADSCs is a physiologically important procedure for regeneration of cartilage tissues. In order to precisely mimic chondrogenesis, a series of engineering approaches to develop the optimal conditions for ADSC expansion have been studied [16, 17]. One of the important criteria for standardized clinical process for ADSC expansion is the composition of cell culture media to enhance proliferation without any deformation in genetic and immunogenic characteristics of ADSCs. In particular, it is suggested that composition, concentration and type of serum components should be optimized to facilitate both proliferation (during the expansion) and differentiation of ADSCs (after transplantation) [18].

Fetal bovine serum (FBS), a mixture of various hormones, growth factors (GFs), antibodies and unknown protein components, is a major serum component in various cell culture experiments to keep maintaining cellularity. A dramatic price increase due to the limited supply and availability [19] may hinder manufacturing of autologous/allogenic cellular products in a large quantity. Therefore, several alternative serum components have been evaluated as FBS substitutes, and these substitutional serums were tested especially for ADSC expansion in a large quantity [20, 21]. Bovine serum (BS, Calf serum) or newborn calf serum (NCS), which can be produced when the calves are 16 months and around 10 days, respectively, have been used as FBS substitutes [22]. Some studies have been conducted to regenerate cartilage tissue using chondrocytes cultured in BS or NCS containing media [23, 24].

In order to evaluate the possibility of several serums as a FBS substitute during stem cell expansion, the level of chondrogenic differentiation of progenitor cell population in different serum conditions should be investigated. For substitute FBS, another serum must assure cell viability and expansion that are not affect to ADSC. During chondrogenesis period, cell expansion is limited because energy of cells is concentrated on differentiation [25]. To this end, in this paper, the effect of serum types (i.e., FBS, BS, and NCS) on proliferation of ADSCs during in vitro expansion and chondrogenic differentiation capacities were evaluated. During 2 weeks of expansion periods, 10% of volume concentration of each serum type in culture media was applied to 2D cultured ADSCs. Proliferation of ADSCs was evaluated using quantification of isolated double stranded DNA (dsDNA) and Live/Dead fluorescence staining, while in vitro chondrogenic differentiation was quantified using dimethylmethylene blue (DMMB) assay, chondrogenic marker gene expression profiling via real-time PCR (RT-PCR), and alcian blue staining.

## Methods

### ADSC expansion and culture

**Scheme 1:**
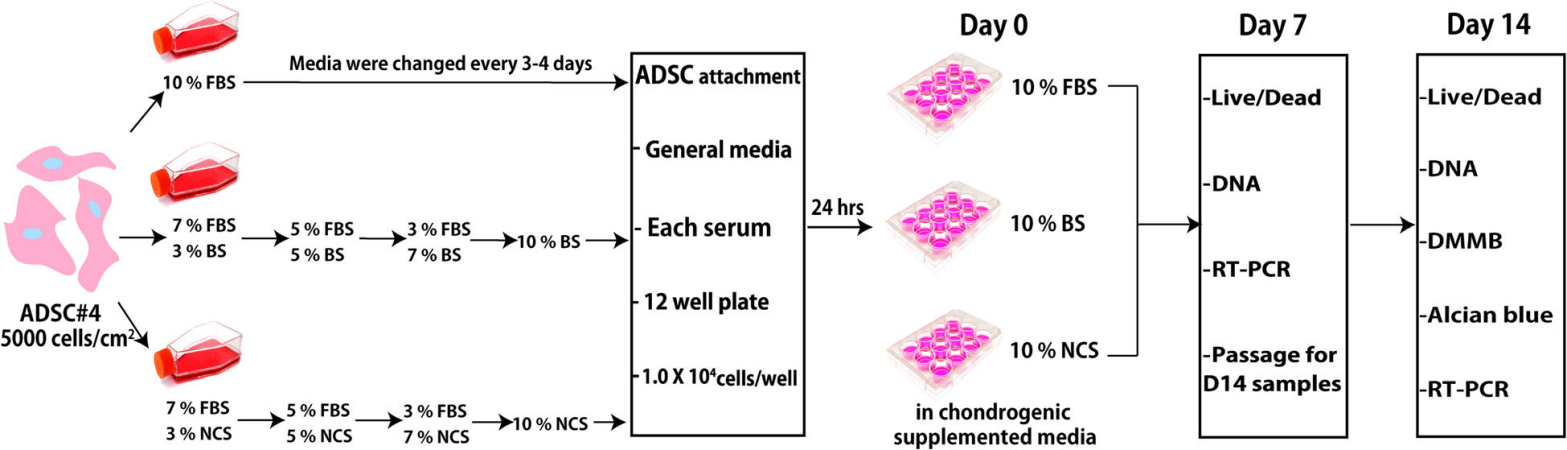
Schematic procedures for serum adaptation and expansion of ADSCs in a chondrogenic culture condition

**Fig. 1:**
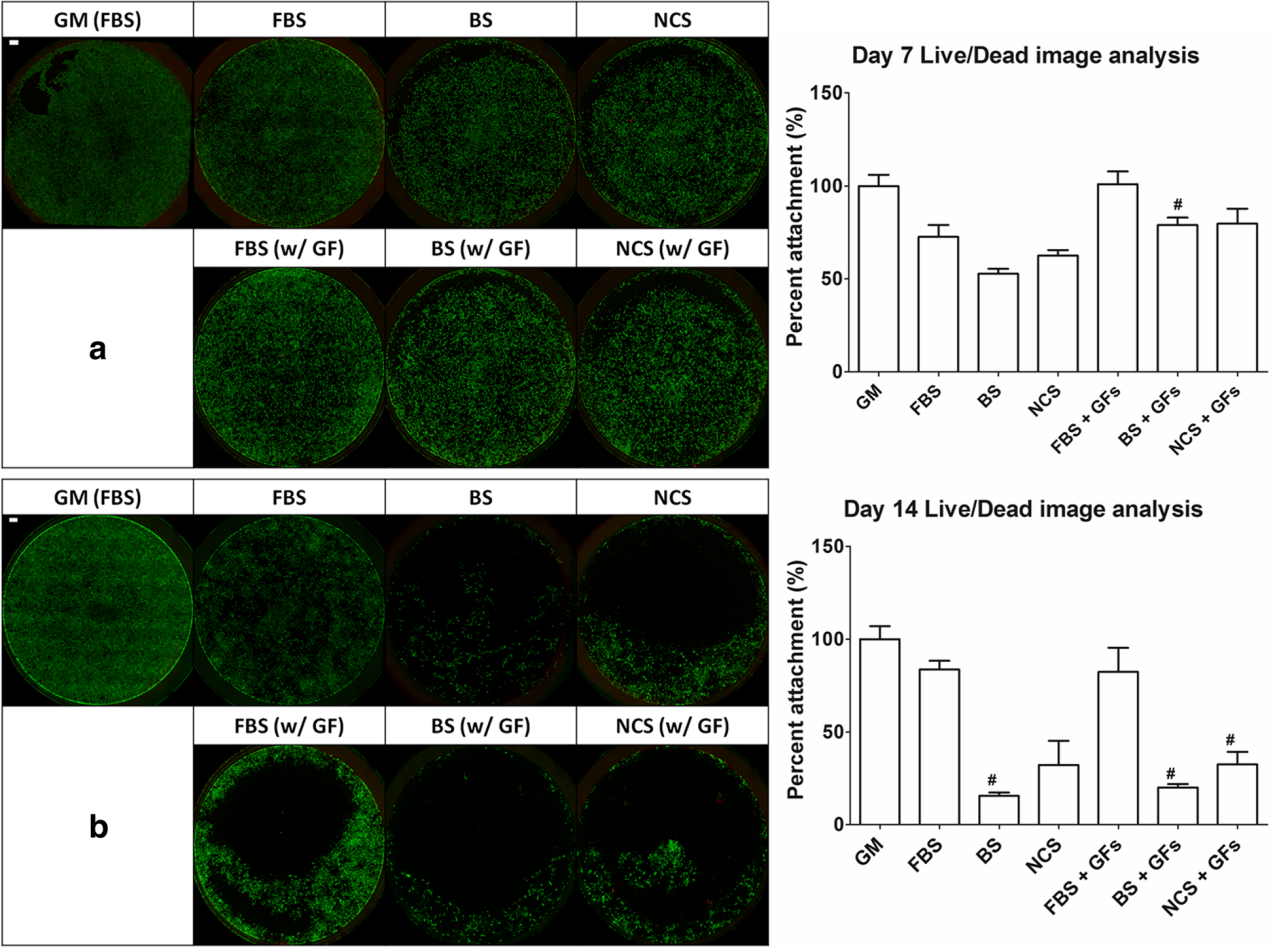
Viability and distribution of ADSCs stained using Live/Dead assay on day 7 (**a**) and day 14 (**b**) in different serum and growth factor conditions. Scale bar = 1000 μm

ADSCs were purchased from Lonza (Walkersville, USA), and pre-cultured using L-DMEM (Wisent, Quebec, Canada), 1% penicillin/streptomycin (Wisent), 7% FBS (Corning), and 3% BS (Gibco) or 3% NCS (Gibco) in T-25 culture flasks up to passage number of 3 over 14 days. Overall scheme of ADSC expansion and culture is described in Scheme 1. For serum adaptation, the volume percentage of FBS was gradually reduced to 5, 3, and 0% (with increasing volume percentage of BS or NCS from 5, 7, and 10%) by changing media every 3 days, while 10% FBS was used as a control (depicted in Fig. 1). Once 80% confluency of cell layers was obtained, ADSCs were then re-seeded at 10,000 cells in each well of 12 well plates with media containing 10% of each serum. After 24 h, media was exchanged to chondrogenic-supplemented media, composed of h-DMEM, 100 nM dexamethasone, 0.05 g/L ascorbic acid, 1% Insulin-transferrin-selenium (ITS) + pre-mix, 3.7 g/L sodium bicarbonate [26, 27], and 10% FBS or NCS or BS. Additionally, to investigate the effect of exogenous GFs on facilitated chondrogenic differentiation of ADSCs, 100 ng of insulin-like growth factor-1 (IGF-1) and 10 ng of transforming growth factor-β3 (TGF-β3) [28, 29] were added into each serum-containing chondrogenic media. A detailed description for experimental groups is demonstrated in Table 1. Seeded cells were cultured at the standard culture condition (37 °C and 5% CO_2_) for the next 14 days. Media was changed every 3 days, and cells were passaged into two separate wells in 12-well plate on day 7.

**Table 1:** Experimental group

Group	GM (FBS)	FBS	BS	NCS	FBS (w/GF)	BS (w/GF)	NCS (w/GF)
Culture Media	General Media	Chondrogenic Supplemented Media			Chondrogenic Supplemented Media		
Serum type	FBS	FBS	BS	NCS	FBS	BS	NCS
Growth Factor	No addition				10 ng/mL of TGF-β3 and 100 ng/mL of IGF-1		

Experimental group was divided into 6 groups by varying serum and presence of growth factors

### Viability & Proliferation of ADSCs

To examine viability and distribution of ADSCs, Live/Dead fluorescent staining (Invitrogen) was performed on day 7 and 14. Any non-adhesion cells were completely removed by a PBS washing (2 times) before the staining process. After washing steps, cells were stained with 2 μM of calcein AM and 4 μM of ethidium homodimer-1. Stained cells were examined using an inverted fluorescence microscope (Ti-E System, Nikon, Japan). Stained area with calcein AM (i.e., area covered by live cell population) was quantitatively analyzed using ImageJ software. % cell attachment was calculated by “a total stained area of each group/a total stained area of GM group × 100 (%)”. To quantify the proliferation of ADSCs in different serum condition, isolated double stranded DNA (dsDNA) amount was evaluated using Picogreen fluorescence kit (Thermo scientific). After washing cell layers with PBS twice, 100 uL of RIPA buffer (Elpis Biotech, Korea) was added in each well. After destruction of the cell monolayer by scraping it using micropipette tips, a suspension containing isolated-dsDNA was collected into a sterile microcentifuge tube. Quant-iT PicoGreen dsDNA Assay Kit (Thermo Scientific) was used for the quantification of dsDNA contents, following the company’s protocol. Fluorescence intensity of each sample (*n* = 3) was recorded at 480 nm of excitation and 520 nm of emission using a microplate reader, and dsDNA amount was calculated using a lambda DNA standard curve.

### DMMB assay

To evaluate chondrogenic differentiation of ADSCs, dimethylmethylene blue (DMMB) assay was performed to quantify glycosaminoglycan (GAG). GAG is one of the characteristic extracellular matrix (ECM) molecules existed abundant in cartilage tissues. Cells were lysed using RIPA buffer by the same protocol for dsDNA isolation. DMMB with pH adjusted to 3.0 was used to quantify total sulfated GAG in each suspension against a standard curve of chondroitin-4-sulfate and L-cysteine hydrochloride. Optical density of each sample was recorded at 520 nm using a microplate reader. The final GAG contents were normalized using corresponding DNA amount (*n* = 4) [30, 31].

### Alcian blue

Alcian blue staining was also used to visualize cartilage ECM deposition by staining GAG contents in chondrogenically differentiated cells. After washing cells with PBS twice, cells were fixed using 4% pafaformaldehyde (Sigma-aldrich) for 30 min. After washing the samples 3 times to remove any remaining reagents, alcian blue solution (pH 2.5, Sigma-aldrich) was added and incubated for 1 h at room temperature. After a removal of staining reagents from each well, images of stained ADSCs were obtained using a camera-equipped optical microscope (Ti-E System, Nikon, Japan) [32].

### Real-time polymerase chain reaction

To analyze gene expression profiles during chondrogenic differentiation of ADSCs, expression of some characteristic marker genes including collagen type II (Col 2), aggrecan (Agg), Sox-9, and collagen type I (Col 1) was evaluated using real-time PCR. GAPDH was used as a housekeeping gene. Primer sequences for genes are listed in Table 2. Total RNA was isolated from trypsinized/pelleted ADSCs with RNeasy Mini Kit (Qiagen) following company’s protocol and prepared in Nuclease-free water (Affymetrix, Inc., Cleveland, Ohio, USA). Both quantity and quality of isolated RNA samples were evaluated with A260/A280 ratio by using Nanodrop (Thermo scientific). Then, cDNA was synthesized with 100 ng of RNA templates using ReverTra Ace qPCR RT Master Mix (Toyobo, Japan). Next, each cDNA template were mixed with SYBR Green Master Mix (Toyobo, Japan), and PCR was performed (*n* = 3) using StepOnePlus Real-Time PCR System (Applied Biosystems). The results were analyzed by using 2^-ΔΔ^C_t_ methods [33, 34].

**Table 2:** Primer sequence for RT-PCR

Gene	Forward(5′- > 3′)	Reverse(5′- > 3′)
Col I	CTC CGG CTC CTG CTC CTC TTA	GCA CAG CAC TCG CCC TCC C
Col II	ATA AGG ATG TGT GGA AGC CG	TTT CTG TCC CTT TGG TCC TG
Aggrecan	TTG AGC AGT TCA CCT TC	CTC TTC TAC GGG GAC AGC AG
Sox-9	CCC AAC GCC ATC TTC AAG G	CTG CTC AGC TCG CCG ATG T
GAPDH	GGG AGC CAA AAG GGT CAT CAT CTC	GAG GGG CCA TCC ACA GTC TTC

Primer sequences for RT-PCR using chondrogenic gene markers. Collagen type II, Aggrecan and Sox-9 were used for chondrogenic markers with using collagen type I as a negative gene marker. GAPDH was used as a housekeeping gene

### Statistical analysis

DNA, DMMB assay and RT-PCR were performed with independent triplicates. Statistical analysis was performed using GraphPad PRISM software (GraphPad software Inc., San Diego, CA, USA). All data were analyzed by one-way analysis of variance (ANOVA) and Tukey’s multiple-comparison test. The means and standard deviations were presented in the Figs. A statistical significance was considered when *p* < 0.05.

## Results and discussion

### Morphology and viability of adipose-derived stem cells

To confirm cell distribution of ADSCs, Live/Dead fluorescence staining was performed at day 7 and 14 (Fig. 1). On day 7, it is hard to distinguish the morphological differences and viability of each group as compared to the control (i.e. cells in growth media with FBS). Decent numbers of ADSCs were viable over 7 days of in vitro culture in any serum conditions regardless of the presence of chondrogenic GFs including IGF-1 and TGF-β3. Image analysis showed in BS containing GF groups showed lowest intensity compared with another group (Fig. 1a). However, on day 14, both parameters including serum types and chondrogenic GFs influenced the attachment of ADSCs (Fig. 1b). Specifically, in ADSCs cultured without GFs, attachments of ADSCs with BS and NCS were far less than those with FBS. Same observation was found in ADSCs cultured with chondrogenic GFs and image data analysis data. As compared with cells with FBS, other serum substitute groups exhibited less cell attachment. Although ADSCs were gradually exposed and adapted to other FBS substitute serums during a pre-culture period, a long-term exposure of different serum types might negatively affect ADSC's attachment.

### Proliferation of adipose-derived stem cells

**Fig. 2:**
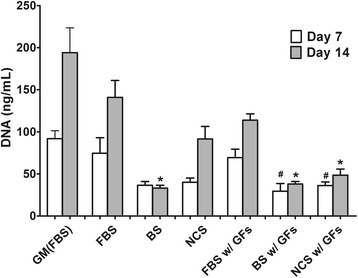
Proliferation of ADSCs, determined using dsDNA amounts, on day 7 and 14 (*n* = 4). # indicates a significant difference (*p* < 0.05) as compared with FBS group within the same growth factor condition on day 7 while * indicates a significant difference (*p* < 0.05) as compared with FBS group within the same growth factor condition on day 14

To analyze proliferation of ADSCs, PicoGreen DNA assay was utilized on day 7 and 14 in quadruplicates. As compared with the control, all groups with chondrogenic supplemented media exhibited less proliferation over 14 days, regardless of serum types and additional GFs (Fig. 2). ADSC proliferation using DNA assay exhibited a similar pattern in the image analysis of live ADSC attachment in Fig. 1. Cells cultured with FBS (both FBS and FBS w/ GFs) showed an increase in proliferation for 14 days. However, BS groups showed the least proliferation, indicated by no significant difference between day 7 and 14 regardless of GF conditions as shown in the image analysis in Fig. 1. Although NCS groups exhibited an increase in proliferation after day 7, the proliferation of ADSCs was highest in FBS groups regardless of the presence of additional GFs. These observations were correlated with the attachment patterns in Fig. 1, which also indicated the highest level of ADSC adhesion in FBS groups, an intermediate level in NCS groups, and the least attachment in BS groups regardless of additional GFs. Therefore, in terms of cellularity and proliferation of ADSCs, it could be reasonably speculated that (1) the usage of BS or NCS as FBS substitutes might not achieve the same level of ADSC proliferation over 14 days of in vitro expansion regardless of additional exogenous IGF-1 and TGF-β3, and (2) the usage of chondrogenic supplemented media might reduce proliferation as compared with the control group of FBS-containing general growth media.

### Chondrogenic gene expression profiles

**Fig. 3:**
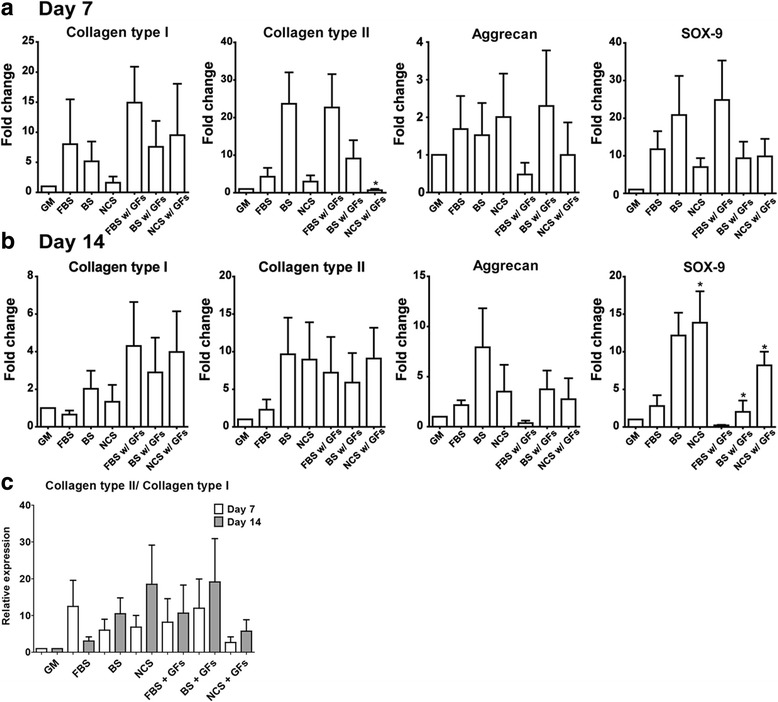
Chondrogenic marker gene expression using RT-PCR on day 7 (**a**) and 14 (**b**) (*n* = 3). The relative expression ratio of collagen type II to collagen type I was presented in (**c**). * indicates a significant difference (*p* < 0.05) as compared with FBS group within the same growth factor condition

To confirm chondrogenic differentiation of ADSC for 7 and 14 days, RT-PCR was performed to analyze chondrogenic gene expression in triplicate. Expression levels of collagen type II, aggrecan, and Sox-9 were evaluated while collagen type I was used for negative chondrogenic gene and GAPDH for endogenous control gene (Fig. 3). At day 7, without additional GFs, BS group showed higher expression of collagen type II, aggrecan, and Sox-9 (Fig. 3a). However, these expression levels did not show significant difference between serum types. When additional GFs were applied, FBS group exhibited relatively higher expression in collagen type II and Sox-9 without significance. Similar trends were observed in day 14 profiles (Fig. 3b). At day 14, in collagen type II, none of serum groups without exogenous GFs showed a higher level expression than control. Only BS w/ GFs group exhibited higher collagen type II expression than control, but without a significance. Similarly, BS groups showed higher expression level as compared with other serum types regardless of additional GFs. However, no significant difference was observed. In Sox-9 expression, NCS groups showed higher expression level as compared with other serum types regardless of additional GFs as well as the control, without a significance. The expression ratio of collagen type II to collagen type I also indicated no significant difference between serum types within the same GF condition (Fig. 3c). Although BS group showed a higher ratio as compared with FBS or NCS in both cases of additional GF condition, the lack of significance might lead no beneficial influences of serum substitutes used in terms of chondrogenic differentiation of ADSCs.

### Quantification and histological staining of glycosaminoglycan

**Fig. 4:**
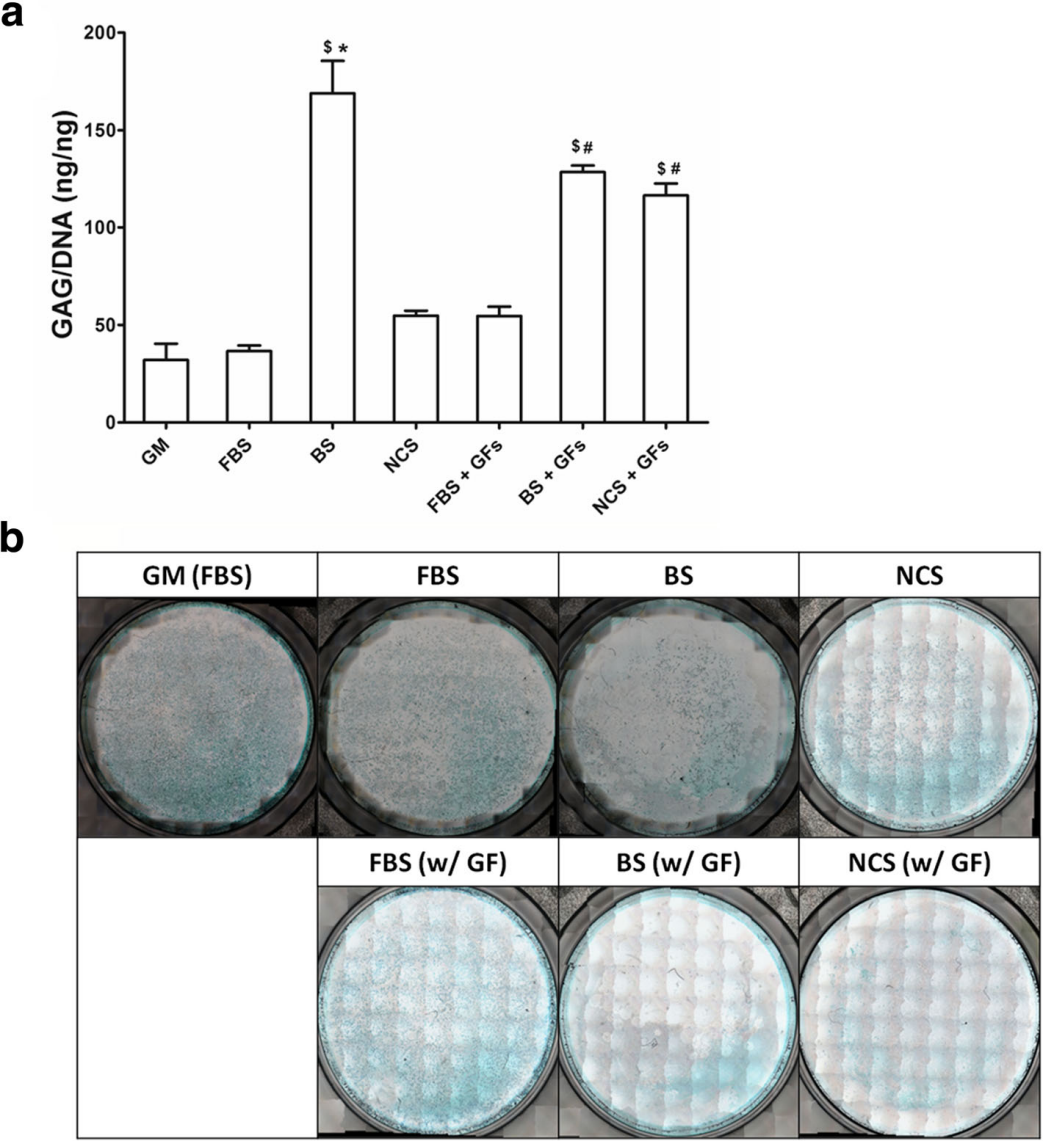
GAG deposition (**a**) and histological observation of alcian blue staining (**b**) on day 14. $ indicates a significant difference as compared with GM group. * indicates a significant difference as compared with FBS group within the same growth factor. # indicates a significant difference as compared with FBS group with growth factors. (*^$#^*p* < 0.05)

To evaluate chondrogenic differentiation of ADSCs on day 14, DMMB assay was used for quantification of GAG contents (Fig. 4a). In the absence of additional GFs, BS groups exhibited a significantly higher normalized GAG deposition as compared with other serum types of FBS and NCS. When exogenous GFs were added, both BS and NCS groups (BS w/GFs and NCS w/GFs) showed a significantly higher GAG level than FBS w/GFs group. BS has a lot of hormones and GFs than other serums, so unknown factors enhanced chondrogenesis regardless of additional GFs. Bovine serum’s differentiation abilities without exogenous GFs were shown in previous study showed that bovine serums can differentiate myogenic satellite cell into myotube or adipocyte-like cells [35]. Although a higher relative expression ratio of collagen type II to collagen type I in BS and NCS groups regardless of additional GFs (Fig. 3c) did not show significant differences as compared with FBS groups, GAG deposition measured by DMMB assay directly demonstrated that BS and NCS could promote chondrogenic differentiation of ADSCs.

Histological examination using alcian blue staining also indicated chondrogenesis of ADSCs in Fig. 4b. Blue-color stained GAG deposition seemed to more intensively appear in FBS groups, however this observation could be related with cellularity per well. As shown in Fig. 1b, the total number of ADSCs in FBS groups with or without GFs was higher than other serum groups. Therefore, the stained GAG area in Fig. 4b could be larger due to the increased proliferation level. However, the normalized GAG contents by DNA amount in Fig. 4b could be more relevant to describe the effect of serum types on chondrogenic differentiation of ADSCs, especially when cultured in chondrogenic supplemented media in the present study. This result might suggest a possibility of using BS or NCS as FBS substitutes specifically when ADSCs were expanded in chondogenic culture condition including stimulant chemicals as well as additional IGF-1 and TGF-β3.

Furthermore, these serums can be used to induce various differentiations such as osteogenesis, neurogenesis and cardiac differentiation in addition to chondrogenesis like previous studies [36–38]. However, one previous investigation reported that anti-adipogenic protein components in BS (i.e., alpha-2-macroglobulin and paraoxonase/arylesterase 1) could inhibit the differentiation of 3T3-L1 into adipocyte [39]. Therefore, it is recommended that a FBS substitute should be specifically selected with caution to optimize the desired control of cellular differentiation. It is of importance to optimize the media composition for a large expansion and cultivation of MSC populations, especially in pharmaceutical industries. A variety of chemical and biological components have been used in the medium to maintain MSC proliferative capacity as well as stimulate the differentiation into desired phenotypes. In addition to additional stimulants in the culture media, a serum is another critical component to design the optimal media composition. For the development of ADSC-based cell therapy products, both proliferation and differentiation capacity of progenitor cell population should be maintained during in vitro or ex vivo expansion. In general, FBS has been used in numerous cases in order to initiate cellular attachment and facilitate a proper proliferation of stem cell population. Although this specific serum type should be inevitably included in the culture media, several studies demonstrated that serum-free condition could be more favorable for stimulating chondrogenic differentiation of stem cells [40, 41]. Moreover, other serum types including BS and NCS have also been tested to substitute FBS due to a high price and currently limited supply of FBS [28, 42]. Treatments of TGF-β3 and IGF-1 have been proved that could induce chondrogenic differentiation of stem cell in serum-supplement condition [43–45]. Based on these results, co-treatment of GFs was applied with various serums. Therefore, in the present study, we evaluated the stimulating effect of serum substitutes of BS and NCS on both proliferation and in vitro chondrogenic differentiation of human ADSCs, as compared with a conventional FBS-containing culture condition. In order to maintain a chondrogenic culture condition, several biochemical components including dexamethasone, ascorbic acid, ITS+ pre-mix, and sodium bicarbonate were added in the media while IGF-1 and TGF-β3 were applied as GF stimulants, in this present study. The result in ADSC proliferation (Fig. 2) demonstrated that a cellularity over 14 day could be more enhanced in FBS-containing conditions as compared with other serum substitutes. In addition, the proliferation of ADSCs in the chondrogenic condition was less than that in FBS-containing general media. This limited proliferative capacity in chodrogenic environments was also observed in a series of studies [46]. In terms of chondrogenic differentiation of ADSCs, it could be anticipated that a serum type might influence the changes into chondrogenic phenotypes of ADSC population, as observed in in vitro GAG deposition in Fig. 4. Although chondrogenic gene expression levels did not show any significant changes in differentiation by modulating serum types as well as additional GF supplements, our result presumably demonstrated that a composition of chondrogenic media (i.e., serum types and presence of additional GFs) could control in vitro chondrogenesis of ADSCs, as compared with FBS-supplemented conditions. In addition to other controlling parameters to enhance cellular expansion and chondrogenesis of stem cells including hypoxia [47]**,** seeding density [48], or culture temperature [49], the use of FBS substitutes including BS and NCS could also be a consideration for obtaining an optimal chondrogenic differentiation of ADSCs.

## Conclusion

Nowadays, a number of studies have investigated the effect of serum substitutes to replace FBS in order to reduce the manufacturing costs for a large production of ADSC-based cellular products. To this end, it could be informative to provide experimental outcomes to compare biological influences of a series of FBS substitutes including BS and NCS. Our results demonstrated that two controlling parameters of serum types and exogenous supplements of GFs could influence in vitro proliferation and chodrogenic differentiation of ADSCs: (1) proliferation of ADSCs was more facilitated in FBS condition, (2) chondrogenic phenotypes evaluated by marker gene expression were not significantly influenced, and (3) cartilage ECM deposition (i.e., GAG contents) was more enhanced in BS condition. Taken together, serum types and exogenous supplements of GFs could be also considered to determine the optimal culture media composition, especially for enhancing linage specific ADSC chondrogenic differentiation during expansion periods.

## Abbreviations

ADSC: Adipose-dervied stem cell
BS: Bovine serum
ECM: Extracellular matrix
FBS: Fetal bovine serum
GAG: Glycosaminoglycan
GF: Growth factor
IGF: Insulin-like growth factor
NCS: Newborn calf serum
MSC: Mesenchymal stem cell
TGF- β: Transforming growth factor
